# Stripping of PFA Fluoropolymer Coatings Using a Nd:YAG Laser (Q-Switch) and an Yb Fiber Laser (CW)

**DOI:** 10.3390/polym11111738

**Published:** 2019-10-24

**Authors:** Guillermo Guerrero-Vaca, Óscar Rodríguez-Alabanda, Pablo E. Romero, Carlos Soriano, Esther Molero, Jon Lambarri

**Affiliations:** 1 Department of Mechanical Engineering, University of Cordoba, Medina Azahara Avenue, 5, 14071 Cordoba, Spain; guillermo.guerrero@uco.es (G.G.-V.); esther.molero@uco.es (E.M.); 2 IK4-Tekniker, Advanced Manufacturing Technologies Unit, Iñaki Goenaga Street, 5, 20600 Eibar, Spain; carlos.soriano@tekniker.es (C.S.); jon.lambarri@tekniker.es (J.L.)

**Keywords:** laser stripping, laser coating removal, PFA coatings, Nd:YAG laser Q-switch, fiber laser CW

## Abstract

Fluoropolymers such as PFA are used as coatings for the protection of metal substrates due to their high chemical inertia and non-stick properties. These are “wear and tear” coatings and they degrade, at which point they should be removed for a new application. The removal of these types of coating by laser is of interest due to the process’s flexibility, precision, ease of automation, and environmental sustainability. The efficiency of the procedure was shown with the use of a source in a pulsed Nd:YAG and a source in continuous mode of fiber (Yb). The rates of stripping (cm^2^/min) and fluence (J/cm^2^) were analyzed and related to the power of the laser sources. Variations of the substrate after stripping were studied: roughness and hardness. The properties of the coating, thickness, roughness, water sliding angle, and microhardness were also evaluated. It was concluded that the laser in continuous mode was more efficient than the pulsed laser; laser removal of fluoropolymers has a strong relationship with reflectivity, and the mechanical and surface properties of the substrate after stripping remained virtually unchanged.

## 1. Introduction

The laser radiation cleaning process has become a solid alternative to the more traditional methods of thermal, mechanical, and chemical nature in a wide range of materials and applications, such as removal of polymeric coatings [1,2,3,4,5]; cleaning of art works [6], antiquities, and buildings [7,8]; nuclear and biological decontamination [9]; mold cleaning [10]; and particle removal in the microelectronics and optics industries [11].

The advantages over current technologies include, in addition to those typically applicable to all laser processes (precision, flexibility, ease of automation), two main important aspects. One of them is the sustainability of the process from an environmental point of view [12], due to the fact that the use of potentially toxic and polluting organic solvents, such as those used in techniques based on chemical removal, is eliminated.

On the other hand, there is the fact that lasers offer the possibility of dealing with new problems, unapproachable by alternative methods. A typical example is the removal of nanometer-sized silica particles deposited on silicon wafers [13]. Metal surfaces are particularly suitable for many laser cleaning and stripping applications. Coatings, residues, or oxides to be removed are affected while the laser ablation stops at the substrate due to reflection on the metal surface. The power density of the laser beam can be easily adjusted to achieve the desired result. This is an important advantage compared to other conventional methods.

In addition, vaporized materials can be captured in the same zone of their emission through a smoke extraction system [14], eliminating the possibility of contaminant emissions in the work area and the need for a subsequent cleaning stage. As for the nature of the laser, the vast majority of published works employed pulsed sources with nanosecond regimes [15,16,17]. Longer pulses induce deeper thermal effects on the substrate, and the risk of damaging the material is therefore greater. Shorter pulses, i.e., peak and femtosecond, may work in some cases, although, due to their reduced average power, they are not suitable for industrial use.

Two clearly differentiated fields have been developed under the umbrella laser cleaning: the removal of particles and the removal of solid surface layers [18]. In the framework of this second category, other processes of laser stripping or separation could also be included if not only the removal of contaminant layers is considered, but also other related processes, such as degreasing, removal of oxides, or stripping of paint and polymeric coatings. The latter is the case in this work.

In this study, we tackled various aspects related to the removal of a PFA (perfluoro alkoxy alkane) fluoropolymer coating by means of two types of laser source that were applied on an EN AW5754 aluminum–magnesium substrate.

PFAs are a highly fluorinated polymer. They are copolymers of tetrafluoroethylene (C_2_F_4_) and perfluoro ethers C_2_F_3_OR^f^, where R^f^ is a per fluorinated group such as trifluoromethyl (CF_3_). Corporations such as Whitford Company, Du Pont de Nemours & Co, Daikin, Ilag, and Grebe Group produce formulations for PFA coatings.

PFA coatings are widely used for their high chemical inertia, non-stick properties, and on surfaces that must be provided with low wettability. They are commonly used as protection inside chemical reactors [19], in metal parts for the demolding of food dough, and particularly in trays and molds for the baking industry. [20]. The removal of these coatings is complex due to their high adhesion to the substrate and high resistance to relatively elevated temperatures (400 °C).

It is a challenge to develop efficient stripping solutions using techniques other than conventional techniques such as thermal degradation of the polymer in a furnace.

It is known that for other fluoropolymers such as PTFE (polytetrafluoroethylene) and FEP (fluorinated ethylene propylene), laser stripping results have been relatively efficient compared to conventional methods [21]. There is a level of uncertainty in efficiency with PFA coatings. The studies of PFA coatings for laser removal found in the literature have had different objectives. There have been works for stripping electrical cables [22,23,24] and for stripping electronic components [25]. Excimer ultraviolet lasers and titanium–sapphire lasers in the infrared field have been used in cable stripping. A KrF excimer laser of 234 nm was used to clean electronic microcomponents (chips). In our study, two laser sources were selected, one of Nd:YAG in pulsed mode operating at the nanosecond regime, and another of Yb fiber in continuous mode.

Nd:YAG pulsed mode lasers have been used to remove epoxy paint contamination on metallic surfaces [26], to clean contaminants from optical glasses [27], to remove impurities from steel sheets [17], to remove titanium nitride from hard metal [28] or carbon steel [16], to clean stones and historical heritage pieces [29], and to remove graffiti [30], among other uses. These are usually laser sources of power between 1 and 300 W and with fluences of 10 J/cm^2^ or less. This type of equipment is usually selected for delicate operations and thin layers.

The continuous mode fiber (Yb) laser has been used to remove air-drying enamels [4], for the removal of silicone rubber by applying black paint to improve absorbance [31], and for stripping polyester paint on an aluminum substrate [5].

The interaction of the laser with polymeric materials and their ablation mechanisms has been widely studied in recent decades [18,32,33]. When the laser beam hits the coating, part of the laser energy is reflected, part is absorbed, and the remaining energy is transmitted to the substrate. As the temperature rises beyond certain limits in the coating, the fusion, sublimation, vaporization, combustion (carbonization), and decomposition occur by rupture of the joint. This is known as a photothermal or pyrolytic process [34]. On the other hand, the UV laser energy absorbed in the electronic excitation can be dissipated in the form of heat, leading to photothermal decomposition or causing the direct rupture of the coating’s molecular bond, which is classified as a photochemical or photolytic process.

It is also known that lasers operating with ns or lower pulses can generate very high intensities (10^5^ kW/cm^2^ up to GW/cm^2^ or more). This can produce non-linear processes such as multiphotonic absorption, dissociation, and ionization [35]. Through the multiphotonic ionization process, a plasma is produced on the paint surface [36]. The plasma can heat the paint layer by thermal conduction. The plasma expands at a high velocity and produces a strong back pressure on the surface of the coating through a shock wave. This procedure is known as photomechanical ablation [3,37,38].

The two selected lasers allowed determination of the reaction of the PFA polymer to stripping by means of photothermal ablation or pyrolysis and, on the other hand, photomechanical ablation. The authors found it relevant to study stripping with two lasers with different principles and functions.

Finally, it can be indicated that the objectives of the work were: (i) to determine the technological variables needed to optimize the stripping efficiency for the proposed lasers, (ii) to analyze and establish the laser stripping mechanisms, (iii) to determine the surface characteristics and mechanical properties of the substrates in the different phases of the stripping process, (iv) to study the properties of the PFA polymer and establish the influence on the stripping efficiency, and (v) to finally determine the characteristics of the final cleaning treatment to eliminate stripping residues from the substrates.

## 2. Materials and Methods

**Figure 1:**
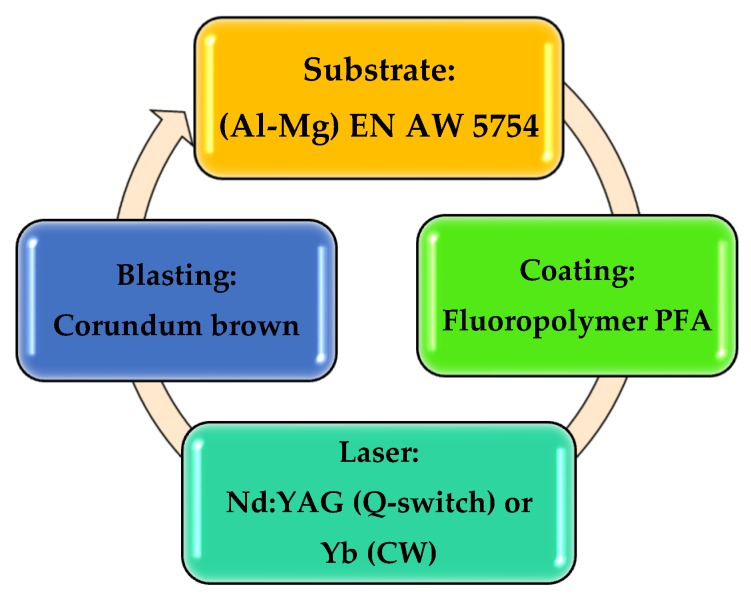
Schematic representation of the perfluoro alkoxy alkane (PFA) laser stripping process presented in this paper.

In order to clarify the information proposed in this section, a sequence of the application process—stripping–cleaning–new application—is detailed in Figure 1. The process is cyclic, i.e., the initial substrate is coated; after use, wear, and loss of properties it is stripped, cleaned, and prepared by blasting with abrasive particles and returns to the initial state. In many parts and supports this cycle can be repeated two to six times.

### 2.1. Substrate and Coating

The substrate used in the experiments was an Al–Mg alloy of type EN AW5754. Twenty samples of aluminum–magnesium alloy 120 × 120 × 1.2 mm^3^ were prepared; two units were left to study the substrate in the state of supply.

The coating used is called TF-77530 by Tecnimacor (Tecnimacor S.L, Cordoba, Spain), a specialist in the application of fluoropolymer non-stick coatings. TF-77530 is a fluoropolymer based on PFA, for which the common uses include: trays and moulds for bakery and related industries, and solutions for engineering in general. It is a two-layer coating. The applied products were supplied by Whitford Company (Whitford España S.L, Barberá del Vallés, Barcelona, Spain). The first was a liquid resin applied by spraying with an HVLP (high volume and low pressure) gun. After drying of the first layer, a second layer was applied by means of an electrostatic powder paint gun. Finally, the whole was cured in an NA 15/65 electric oven (Nabertehem GmbH, Lilienthal, Germany). For color determination, a standard RAL color table (RAL gemeinnützige GmbH, Bonn, Germany) of 1625 colors was used [39]. The coating characteristics are shown in Table 1.

**Figure 2:**
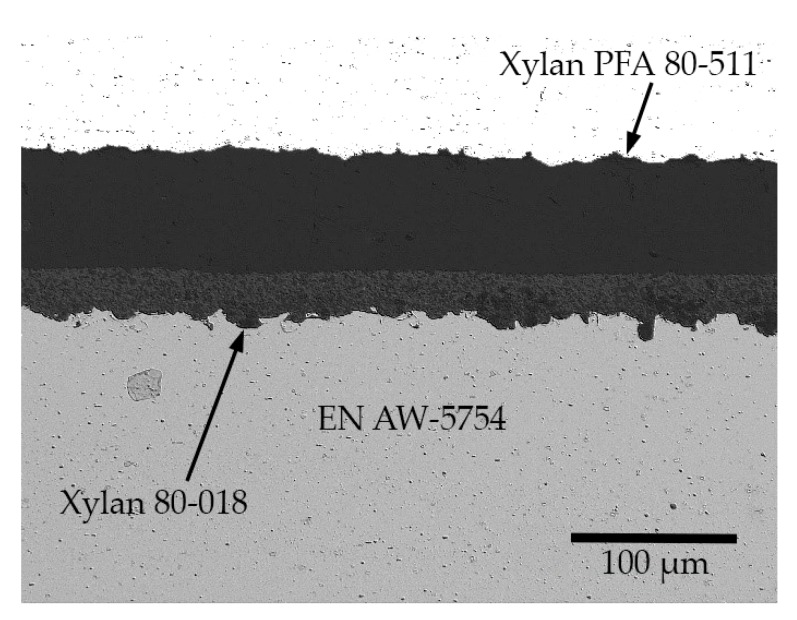
EN AW 5754 substrate with PFA coating (mounting with an aluminum backing plate to preserve the edges).

A micrograph of the cross-section of the aluminum substrate with the applied coating is provided in Figure 2 to show the morphology of the deposited layer.

In addition, the microhardness of the polymer, the angle of surface contact, the fraction of reflected laser light (reflectivity), and surface roughness were measured in the coating. The microhardness of the polymer was obtained using the method of Oliver et al. [40] with a load of 150 mN on a Fisherscope H100 (Fisher Technology Inc., Windsor, CT, USA). The wettability of the coatings was tested with 150 µL Milli Q water droplets on a device equipped with an oscillating platform controlled by an engine at a speed of 0.5°/s and software for digitizing images of the droplet profile [41]. The water droplets used to determine the angle measurement were demineralised and deposited by means of an adjustable volume pipette. A digital inclinometer was used to measure with an accuracy of 0.05°. The inclinometer was magnetic and could be placed on the edge of the tilting platform. The detection of the global drip slip was done visually. For each surface to be analyzed, three drops were deposited and analyzed. The sliding angle was measured as an indicator of non-stick capability [41].

The reflectance values were obtained using a Lambda 950 spectrophotometer (Perkin Elmer, Madrid, Spain) and an integrating sphere to evaluate the fraction of incident energy that was reflected by the coating.

### 2.2. Laser Sources

Two sources with different characteristics were used to study two stripping mechanisms, one dominated by ablation and the other by combustion.

One of the sources was a pulsed Nd:YAG laser (Quanta System S.p.A, Milan, Italy), model Handy Industrale. It is a “Q-switch” laser technology pumped by lamps in the range of nanoseconds. The most remarkable features are a high energy per pulse and a low repetition frequency

The second source was a 200 W (CW) continuous mode fiber (Yb) laser from SPI Lasers (SPI Lasers UK Ltd., Southampton, United Kingdom) air cooled. The characteristics of both sources are given in Table 2.

The Quanta laser had a 200 mm focal length cylindrical focusing lens at the resonator output. An elongated and elliptical spot of approximately 6 × 1 mm was generated over the working area. The specimens were supported on a table to which two linear axes were coupled in order to be able to generate sweeps on flat pieces. A vacuum cleaner close to the working area completed the assembly.

The continuous fiber laser was air-cooled. The radiation was transported to the working area via a 5 m long optical fiber. The laser source was integrated into a three-axis CNC machine. It had a working table of 300 × 300 mm, prepared for the fixation of different specimens and flat components. The table was located on the XY axes of the machine. Each axis had a maximum allowed displacement of 400 mm, with an accuracy of 50 µm. The Z axis has a maximum displacement of 200 mm with a similar accuracy of 50 µm.

The optical path consisted of an 83 mm focal collimation lens together with a 2× beam expander, all connected to a two mirror scanning head. The optical system was completed by a 167 mm focusing lens, which provided a spot of approximately 36 µm in diameter.

**Figure 3:**
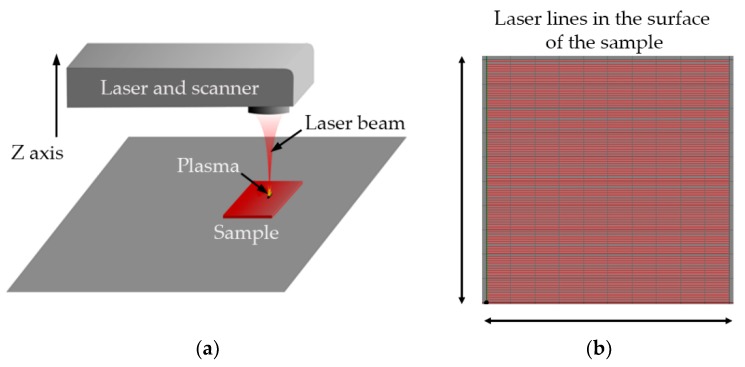
(**a**) Workstation; (**b**) scanner head motion schematic.

The processing strategy was the same for both cases, using a linear sweep to cover the entire area of interest. The lines overlapped a certain distance to obtain a homogeneous treatment, as illustrated in Figure 3. Both lasers were used at the focal position, and no process gas was used.

The maximum available power was set, in both cases, to maximize the stripping rate. The criterion for selecting the operating parameters was to look for the combination of variables that maximized the removal rate. The speed of advance and distance between lines had been determined by previous tests, considering the optical qualities of each laser. In one case, there was an elongated, elliptical spot of 6 × 1 mm and the distances between lines were relatively high, and in the other case a spot of 36 µm in diameter and small distances between lines. Table 3 shows the tests carried out.

### 2.3. Substrates after Stripping

After the laser stripping, the surfaces were characterized from a superficial and mechanical point of view. The roughness Ra, Rz, and Rq were measured with a Mitutoyo roughness meter model Surftest Sj-201 (Mitutoyo Corporation, Sakada, Japan). Study of the micrographic images of the surfaces obtained after stripping were carried out using the JEOL JSM 7800F scanning electron microscope (JEO Ltd., Tokyo, Japan). The hardness of the substrate before and after the laser stripping was measured with a Vickers hardness tester model Zwick/Roell ZHU250 (Zwick Iberica Testing Equipment S.L, San Cugat del Valles, Barcelona, Spain) using a load of 10 kg, according to the UNE-ISO 6507 standard [42].

The microstructure was characterized in the cross section of the substrates through the determination of the percentage of constituent particles (intermetallic) and the measurement of grain size (ASTM). For this purpose, metallographic samples were prepared with cold cured acrylic resin polished with abrasives up to colloidal silica from 0.25 µm. The observations were made with an optical microscope Leica DMI5000 (Leica Microsistemas S.L.U, Hospitalet de Llobregat, Spain) with polarized light. For the analysis of intermetallic particles, the samples were slightly attacked (2 to 3 s) with a solution of hydrofluoric acid in water at 0.05%. Image-Pro Plus (Media Cybernetics Inc, Rockville, USA) analysis software was used for particle counting. The Barker reagent (Struers, Madrid, Spain) with electrolytic anodizing was subsequently used to reveal the granular structure. The grain size was measured by the planimetric method defined in ASTM E-112 [43].

Small traces of polymer were left on the surface of the decoating specimens. For the elimination of these, a projection with abrasive particles was carried out using a Sand Blast Cabinet CAT-990 (Aslak S.L, San Quirze del Valles, Barcelona, Spain). The particles used were brown corundum RBT Gr.60. The projection nozzle had a diameter of 6.5 mm. The projection was carried out at 200 mm from the substrates and with a pressure of 0.4 MPa. The surface roughness was been measured, SEM images were obtained after the final blasting, and the Vickers hardness of the substrates was measured.

## 3. Results

### 3.1. Physical and Optical Properties of Coatings

The roughness of the coatings applied on metallic substrates was determined. Two interesting moments were studied: (i) initial state of the coating before laser stripping and (ii) new application of the coating after laser stripping and cleaning through projection with abrasives. The results are shown in Table 4.

**Figure 4:**
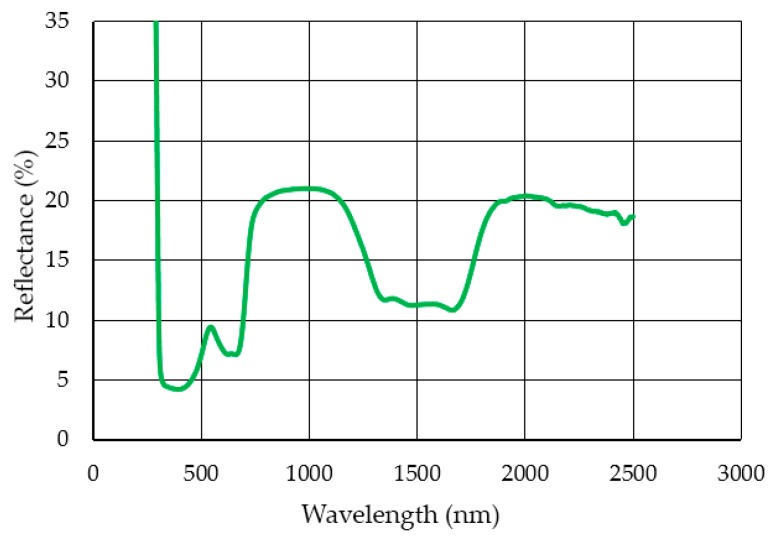
Reflectance values of the PFA coating.

The reflectance of the PFA coating at wavelengths from ultraviolet light, visible spectrum, and near infrared was measured. The results are shown in Figure 4 below.

The reflectance for the lasers studied with wavelengths of 1064 and 1070 nm was 20.89% and 20.84%, respectively—virtually identical values.

Wettability of the coating was studied through the sliding angle (SA) of a 150 µl drop of water before stripping, and once a new coating was applied after stripping. The values were 8.77 ± 0.77 before stripping and 8.37 ± 0.28 after stripping.

**Figure 5:**
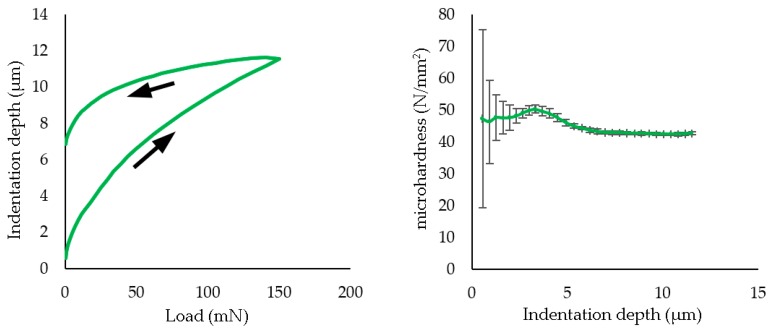
The load–unload curves (**left**) and microhardness (**right**) measured in the PFA coatings.

Microhardness of the coating was determined, and the results are shown in Figure 5. A load of 150 mN was applied in a sequence of 25 stages, with a 1 s interval between each stage.

### 3.2. PFA Stripping with Laser Sources

After having completed and evaluated the stripping trials proposed in Table 3, the best results were obtained in order to maximize the stripping rate. Results are shown in Table 5.

For the nanosecond laser, it was experimentally determined that the ablation threshold was 2.97 J/cm^2^. The combustion threshold was not found, since it was generated for all the explored parameters.

**Figure 6:**
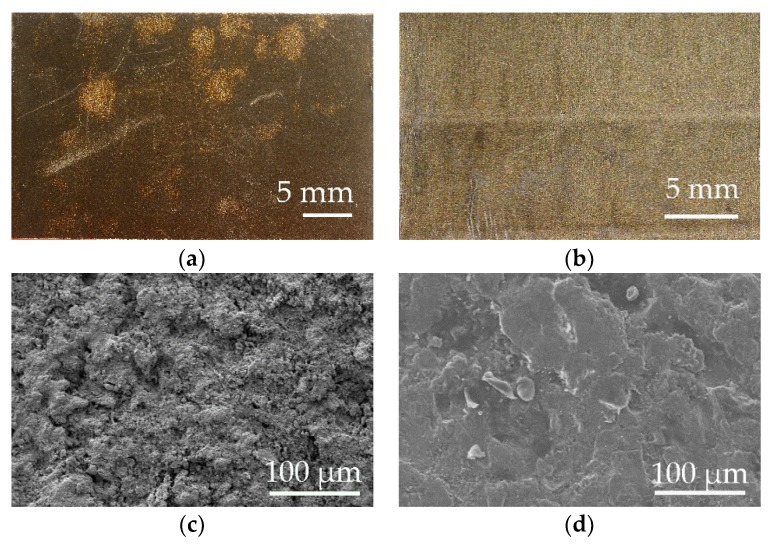
Photography obtained by digital camera and micrography obtained with SEM of the surface appearance of the EN-AW5754 substrate after laser stripping. (**a**) Digital camera SPI laser-CW and (**b**) digital camera Quanta Q-switch, (**c**) SEM SPI laser-CW, (**d**) SEM Quanta-Q-switch.

The appearance of the surface after stripping was evaluated by surface photography and scanning electron microscope (SEM). These images are shown in Figure 6.

Homogeneously distributed pyrolyzed polymer residues were evident in the case of the SPI-CW laser in continuous mode, and unpyrolyzed PFA polymer residues in the case of the Quanta pulsed laser.

After a surface composition analysis by EDX, fluorine was detected at an estimated percentage of 26% in the case of the SPI-CW laser and 13% in the case of the Quanta-Q-switch.

**Figure 7:**
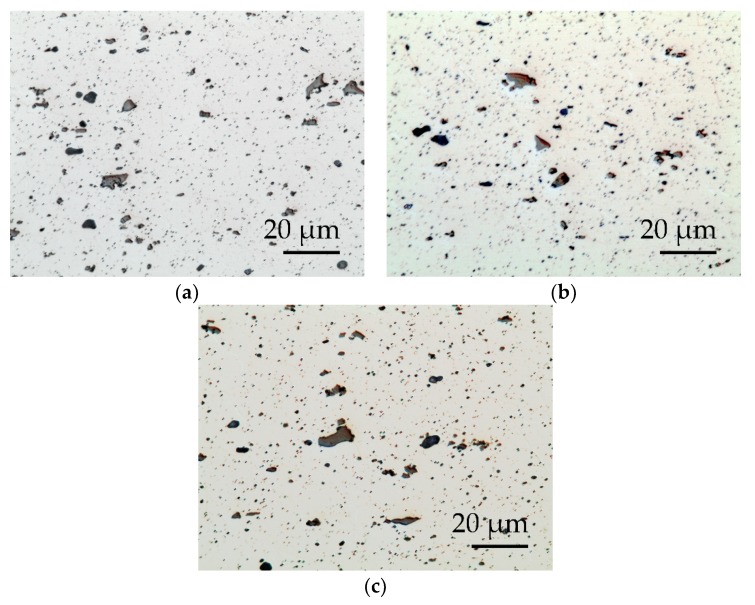
EN-AW5754 metallographic images obtained with colloidal silica polishing up to 0.25 μm, lightly attacked with hydrofluoric acid: (**a**) after PFA coating, (**b**) after Quanta laser decoating, (**c**) after SPI laser decoating.

**Figure 8:**
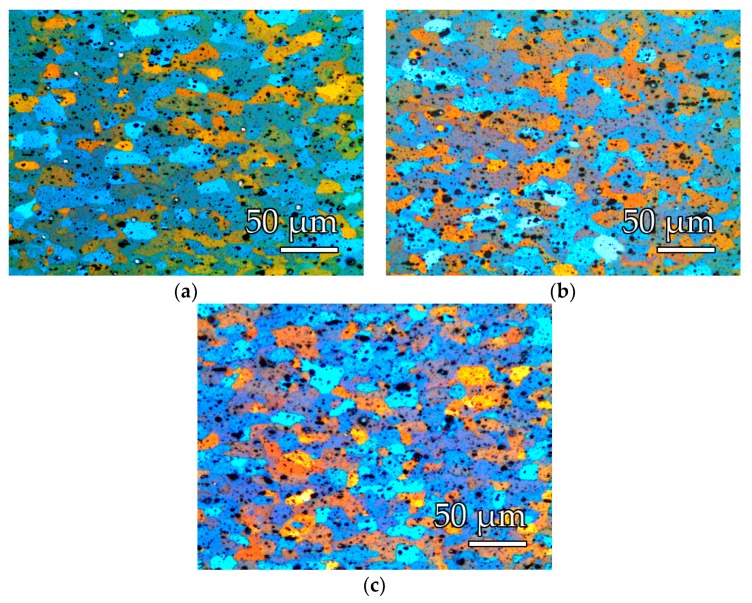
EN-AW5754 metallographic images obtained after polishing and electrolytic anodizing with Barker reagent: (**a**) after PFA coating, (**b**) after Quanta laser decoating, (**c**) after SPI laser decoating.

The images in Figure 7 and Figure 8 show the distribution of the constituent particles in different states and the appearance of the metallographic structure. The substrates of the alloy used AW-5754, after the thermal process of application of the PFA coating, presented a recrystallized structure, formed by crystals of a solid solution α-rich in aluminum and a precipitation of particles (intermetallic), of the types: Al(Fe, Mn)Si and Mg_X_Si (primary precipitates) and Al_6_(Fe, Mn) (secondary precipitates).

Table 6 incorporates the values obtained for Vickers hardness, the % in intermetallic area, and the ASTM grain size.

### 3.3. Condition of the Substrate after Blasting with Abrasive Particles

Final blasting with abrasive particles (brown corundum) was necessary to remove polymer residues in the substrate after laser stripping. In one case, it was eliminated in a shorter time than 5 s to 0.4 MPa (SPI), and in the other case, a time of 20 to 30 s to 0.4 MPa was necessary. In both cases, the superficial roughness, Ra and Rz, were measured. The data are shown in Table 7.

**Figure 9:**
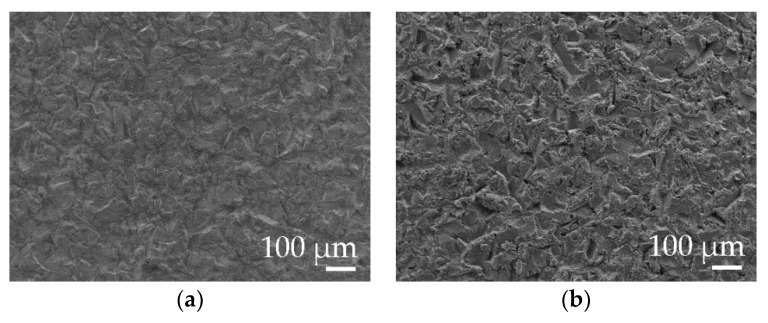
Micrography of the superficial appearance of the EN-AW5754 substrate after blasting with brown corundum at 0.4 MPa (**a**) with SPI laser-CW and (**b**) Quanta-Q-switch.

A photograph obtained in the SEM after the final blasting is also shown. The appearance was indistinguishable after blasting for both laser stripping (Figure 9).

**Figure 10:**
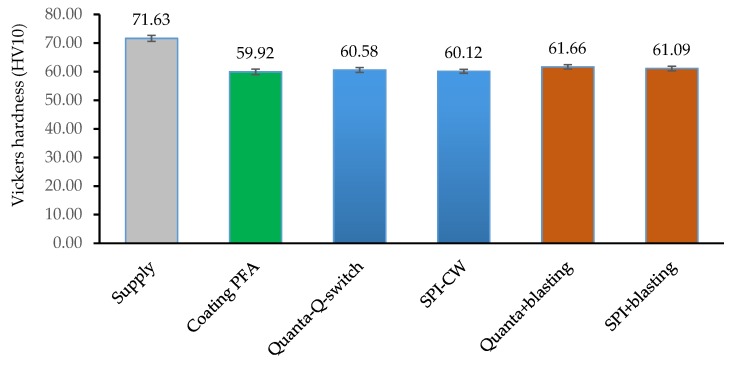
HV10 Vickers hardness on EN-AW5754 substrates in the different phases of supply, PFA coating, laser stripping, and final blasting.

Finally, Vickers hardness was measured on supply, immediately after application of the PFA coating, after stripping, and after the final blasting. The values are shown in Figure 10.

## 4. Discussion

The characteristics of the lasers studied involved different removal mechanisms. On the one hand, the SPI laser is a fiber laser in continuous mode and the mechanism was basically carbonization, photothermal, or pyrolytic. The images in Figure 6 show the appearance after laser decoating. On the one hand, there was a black surface with powdery appearance (Figure 6a) and a SEM image (Figure 6c) that showed a rough and uneven texture. The black remains were weakly anchored to the substrate and could be easily removed manually. In both cases, we obtained the appearance of a coating compatible with carbonization. In addition, EDX analysis of the decoating substrate detected Al, Mg, F, Zn, K, Cr, S, and C, which are compatible with the composition of the substrate, the top layer of PFA coating, and the primer coat.

**Figure 11:**
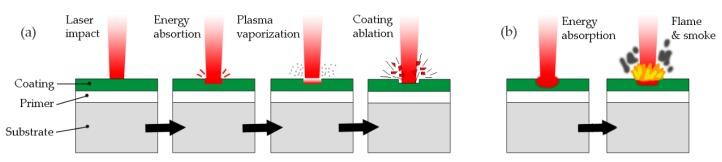
Graphic example of the stripping procedure with a laser source: (**a**) by photomechanical effect, (**b**) by photothermal effect.

On the other hand, the Nd:YAG Quanta model laser that operated in the nano-seconds regime developed a photomechanical ablation phenomenon. The aspect of the striped coating shown in Figure 6b,d indicated that there was no carbonization, and that even traces of fluoropolymer could be seen on the substrate without stripping. Figure 11 shows the phenomenon of photomechanical and photothermal removal.

### 4.1. Laser SPI (CW) and QUANTA (Q-Switch) Efficiency

The laser sources used in the test had very different removal procedures due to the characteristics of the equipment. However, the results were analyzed considering the differences in effective power of each laser: SPI (200 W) and Quanta (10 W).

In the literature [44], results have been obtained for stripping with a Rofin laser source in continuous Nd:YAG mode for fluoropolymers of PTFE (polytetrafluoroethylene) and FEP (fluorinated ethylene propylene). The results are shown in Table 8.

It was evident that there was a higher rate of fluence and decoating with the SPI laser than with the Quanta laser and that this difference was greater than would be expected due to the difference in power only. The relationship between SPI-CW/Quanta-Q-switch was: fluence (30/1), decoating rate (31.5/1), and power (20/1).

PTFE (1.2 cm^2^.min^−1^/W) and FEP (0.187 cm^2^∙min^−1^/W) had the best and the worst ratios between stripping rate and power after the study and from the information the literature. This fact seems to have a strong relationship with the level of laser light reflectivity of each source with the polymer. Lower levels of reflectance produced greater efficiency in stripping. The reflectivity values were: 5–6% (PTFE), 20–21% (PFA), and 28–26% (FEP).

### 4.2. Aluminum Substrate Conditions

After stripping it was necessary to use a blasting process to finish removing carbonized polymer residues or small challenges of PFA, in addition to homogenizing the substrate and preparing it for a new application [19].

This surface treatment produced a final level of Ra between 3–3.5 µm and Rz between 21–25 µm for both types of laser. On the other hand, the type of residue generated on the surface was of a different nature and the blasting time to remove it was: 5 s for SPI-CW and 20–30 s for Quanta-Q-switch. The results of the surface roughness in the aluminum–magnesium EN-AW 5754 substrates showed that there were no differences due to the use of the types of laser source in the study, but that the time needed for the final cleaning was four to six times longer with the Quanta laser than in the SPI. Figure 7 shows that the surface aspects of the substrate were similar.

As far as to the mechanical properties of the substrate are concerned, the Vickers hardness was studied. It is known that due to the curing of the PFA coating in the PFA sintering process, an annealing of the substrate and a notable decrease in hardness occurs [20,21,44]. The decrease was evaluated and is between 13.8–14.6% with respect to the state of supply. A slight subsequent increase in hardness was shown for the Quanta laser versus the SPI. This increase was foreseeable due to the longer blasting time with abrasive particles [45], although in any case it was not very significant at 0.8%.

Compared to the values in the literature, the decrease in hardness was less than in other polymers (PTFE and FEP) and substrates (EN AW5251), from 13.8–14.6% to 35.7% (Table 9). This difference was very predictabe due to the different composition and initial treatment state of the Al–Mg alloy [20].

### 4.3. Coating

After the laser stripping, a new PFA coating was reapplied to the substrate and thus the cycle was closed. It was analyzed whether the modification of the substrate influenced the wettability properties of the deposited coating and, therefore, the non-stick capacity of the coating. The water slide angle was measured. This is an indicator of the wettability level of a surface [46]. The values obtained were 8.77 ± 0.77 before stripping and 8.37 ± 0.28 after stripping. The roughness of the PFA coating was also measured before and after a new application after stripping. There was a slight increase in the value of Ra and Rz. In one case, values of Ra ranged from 0.39 to 0.75–0.65 µm and of Rz from 2.17 to 3.21–3.36 µm. The differences were not very significant, and the response of the coating in terms of its wettability and non-stick capacity seemed to remain unchanged.

The microhardness of the PFA polymer and its thickness were also studied. PFA values ranged from 40–42 N/mm^2^ hardness to 102.3 ± 15.08 µm thickness. The characteristics for other fluoropolymers are known: FEP, hardness 22–30 N/mm^2^ with a thickness 61.3 ± 0.88 µm and PTFE, 62 to 70 N/mm^2^ with a thickness 19.09 ± 0.9 µm [44], and it was deduced that the PFA coating was the thickest applied of the three types and its hardness was intermediate between FEP and PTFE. There did not seem to be a relationship between the hardness of the coating and the laser efficiency of the stripping.

PFA fluoropolymer had the highest thickness of the coatings studied and described in the literature. The thickness ratio between PFA/FEP is 5 to 3 in favour of PFA and in stripping, which could be inferred an inverse ratio, a 2/1 ratio was obtained in favour of PFA for the CW SPI laser. This determines that even with higher PFA coating thicknesses, the stripping efficiency was higher than that of FEP fluoropolymer.

## 5. Conclusions

After analyzing the results, we drew the following conclusions:

- The Quanta laser source (Q-switch), which works with nanosecond pulses, produced the stripping of the PFA coating by photomechanical effect.
- The laser source SPI (CW) produced the stripping of the PFA fluoropolymer by photothermal or pyrolytic effect, in which the polymer was carbonized.
- The Quanta pulsed laser produced the decoating of the PFA coating with an advance of 5 mm/s, a spacing of 6000 μm, and an irradiance of 2.2 × 10^3^ kW/mm^2^ with an elliptical spot of 8 × 0.5 mm.
- The SPI laser (CW) produced the decoating of the PFA with an advance of 5000 mm/s, a spacing of 25 μm and an irradiance of 196 kW/mm^2^ with a circular spot of 18 μm.
- For PTFE, FEP, and PFA fluoropolymers, the highest stripping rate using continuous wave lasers (CW) operating at around 1060–1070 nm was associated with the lowest level of reflectivity. PTFE decoating was the best, then PFA and finally FEP.
- The stripping rate (cm^2^∙min^−1^/W) per power unit was 1.5 higher for the SPI (CW) laser than for the Quanta (Q-switch) laser in PFA fluoropolymers.
- The stripping rate of PFA fluoropolymers by continuous wave laser operating in the 1060–1070 nm environment was higher than that of FEP fluoropolymers, even with 5/3 higher PFA vs. FEP thicknesses.
- The hardness of fluoropolymer PTFE, FEP, and PFA coatings did not appear to influence the efficiency of laser stripping with sources operating in the range of 1060–1070 nm.
- Cleaning by projection of abrasives of the residues produced by the stripping on the substrate required between four to six times longer for the Quanta laser (Q-switch) than for the SPI laser (CW).
- Surface roughness Ra of the substrates after laser stripping and subsequent blasting was at 3–3.5 µm for both types of lasers.
- The Vickers hardness, the percentage of constituent particles, and the grain size between the substrate with the PFA coating and immediately after laser decoating by both sources did not produce significant variations, and showed that the mechanical properties of the substrate remained unchanged between these two states.
- After stripping and cleaning by projection of abrasives there was a slight increase in Vickers hardness (~1%) in the substrate decoating with the Quanta laser (Q-switch) compared to the SPI laser (CW).
- Wettability properties of the PFA coating remained unchanged after a full cycle of application, stripping, and re-application.

## Figures and Tables

**Table 1:** Application process, thickness, and color of PFA TF-77530 coating.

Products (Whitford)	Temperature (°C)/Time (min)	Base Layer Thickness (µm)/Top Layer (µm)	Color RAL
Prymer: Xylan 80-018/G5811	120–150/5	24.6 ± 4.08/75.2 ±14.3	130 40 30 Green
Top: Xylan PFA 80-511/G6003	380/80		

**Table 2:** Laser source characteristics.

Source	Wavelength (nm)	Power (W)	Spot Shape/Radius (mm)	Irradiance (kW/mm^2^)	Pulse Energy (mJ)	Frequency (kHz)	Pulse (ns)
Quanta (Q-Switch)	1064	0–10	Elliptical ^1^/3–0.5	0–2.2 × 10^3^	0–200	0.04	~8
SPI Lasers (CW)	1070	0–200	Circular/1.8 × 10^−2^	0–196.4	-	0–100	-

^1^ Semi-major and semi-minor axes, respectively.

**Table 3:** Values of the explored PFA coating laser stripping tests on EN AW5754 aluminum substrates.

Source	Power (W)	Advance (mm/s)	Line Spacing (µm)
Quanta (Q-switch)	10	1, 5, 7, 10, 20	1000, 2000, 4000, 5000, 6000
SPI Lasers (CW)	200	100, 200, 500, 1000, 2000 3000, 4000, 5000, 6000	10, 15, 20, 25

**Table 4:** Roughness values of PFA coatings applied on EN-AW5754 substrates before and after stripping.

Source	Coating	Roughness (µm)	PFA Initial Coating	PFA Coating after Decoating and Blasting
Quanta (Q-switch)	PFA	Ra	0.39 ± 0.02	0.75 ± 0.04
		Rz	2.17 ± 0.04	3.21 ± 0.05
SPI Lasers (CW)	PFA	Ra	0.39 ± 0.02	0.65 ± 0.03
		Rz	2.17 ± 0.04	3.36 ± 0.05

**Table 5:** Laser stripping trials values in order to obtain the maximum stripping rate.

Source	Power (W)	Advance (mm/s)	Line Spacing (µm)	Fluence (J/cm^2^)	Rate (cm^2^/min)
Quanta (Q-switch)	10	5	6000	4.2	2.4
SPI Lasers (CW)	200	5000	25	110	75

**Table 6:** ASTM grain size, Vickers hardness (HV), and % area of constituent particles of EN AW 5754 alloy in various states.

State	Number of Intercepted Grains	Average Length of Grains Intercepted	Grain-Size ASTM	Vickers Hardness HV10	Particle Area Fraction (%)
PFA coated	1235	0.0159	8.6	59.92 ± 0.41	4.45 ± 0.22
Quanta Q-switch laser decoating	1284	0.0153	8.7	60.58 ± 0.19	4.43 ± 0.37
Laser SPI-CW decoating	1232	0.0159	8.6	60.12 ± 0.11	4.39 ± 0.10

**Table 7:** Roughness values of EN-AW5754 substrates after laser stripping and after brown corundum blasting at 0.4 MPa.

Source	Blasting Time (s)/Pressure (MPa)	Roughness (µm)	Supply	After Stripping
Quanta (Q-switch)	20–30/0.4	Ra	0.25 ± 0.06	3.23 ± 0.24
		Rz	0.87 ± 0.14	23.85 ± 1.09
SPI Lasers (CW)	<5/0.4	Ra	0.25 ± 0.06	3.06 ± 0.36
		Rz	0.87 ± 0.14	21.98 ± 2.01

**Table 8:** Power, process fluence, stripping rate and rate/potency values for PFA, PTFE, and FEP fluoropolymer coatings.

Source	Fluoropolymer	Power (W)	Process Fluence (J/cm^2^)	Decoating Rate (cm^2^/min)	Rate/Power (cm^2^∙min^−1^/W)
Quanta (Q-switch)	PFA	10	4.2	2.4	0.24
SPI Lasers (CW)	PFA	200	110	75	0.375
Rofin (CW) ^1^	PTFE	500	50	600	1.2
Rofin (CW) ^1^	FEP	800	160	150	0.187

^1^ Rodriguez-Alabanda et al., 2019 [44].

**Table 9:** Vickers hardness values (HV10) for PFA-coated EN AW 5754 substrates and PTFE- and FEP-coated EN AW 5251 substrates, in different states.

Source	Substrate	Fluoropolymer	Vickers Hardness (HV10)			
			Supply	Coating	Removal Laser	Sand Basting
Quanta (Q-switch)	EN-AW 5754	PFA	71.6	59.9	60.5	61.7
SPI Lasers (CW)		PFA	71.6	59.9	60.1	61.1
Rofin (CW) ^1^	EN-AW 5251	PTFE	82.3	-	51.8	52.9
Rofin (CW) ^1^		FEP	82.3	-	51.6	51.8

^1^ Rodriguez-Alabanda et al., 2019 [44].
